# Complete Genome Sequences of Two Arthrobacter Phages, assigned to clusters AT and AZ

**DOI:** 10.17912/micropub.biology.001417

**Published:** 2025-01-16

**Authors:** Sarah J. Swerdlow, Melinda Harrison

**Affiliations:** 1 University of Pittsburgh at Greensburg, Greensburg, Pennsylvania, United States; 2 Cabrini University, Radnor, Pennsylvania, United States

## Abstract

Two bacteriophages, BeatusComedenti and Cyan, were isolated using *Arthrobacter sp.* and *Arthrobacter globiformis* , respectively. BeatusComedenti and Cyan, which contain 100 and 70 putative genes, are assigned to actinobacteriophage clusters AT and AZ1, respectively.

**Table 1.** Isolation and sequencing parameters, plaque and genomic characteristics.

**Table:** 

Phage Name	BeatusComedenti	Cyan
Year Discovered	2015	2021
Host	<i>Arthrobacter sp</i>	<i>Arthrobacter globiformis</i>
Soil Sample Collection: Location	Los Altos Hills, CA	Old Westbury, NY
Soil Sample Collection: GPS Coordinates	37.375 N, 122.14 W	40.813 N, 73.61 W
Isolation Method	Enriched	Enriched
Plaque Morphology	Turbid	Turbid
Approx. Shotgun Coverage (fold)	228	833
Genome Length (bp)	58687	43670
G+C content (%)	63.4	67.6
No. of ORFs	100	70
Cluster	AT	AZ1

## Description

Bacteriophages are the most abundant entities on earth and are highly diverse. The isolation and characterization of novel bacteriophages can advance our understanding of virus evolution and inform the development of phage as therapeutics (Hatfull, 2022) . We report here the characteristics of two novel *Arthrobacter* phages, BeatusComedenti and Cyan, that were isolated utilizing *Arthrobacter sp.* and *Arthrobacter globiformis,* respectively (Klyczek et al., 2017; Hatfull 2020) .

The two bacteriophages were isolated from soil in New York and California using standard methods as previously described (Table 1) (Jordan et al., 2014; Zorawik et al., 2024) . These soil samples were incubated in peptone-yeast extract-calcium (PYCa) liquid medium for two hours at 30°C with shaking to suspend phage particles. The suspension was then filtered through a 0.22 µm filter. The filtrate was inoculated with *Arthrobacter sp.* or *Arthrobacter globiformis* and incubated at 30°C with shaking for 2-3 days (enriched isolation) before being filtered and plated, yielding plaques BeatusComedenti and Cyan, respectively (Table 1). Both phages were purified through three rounds of plating. All plates were incubated at 30°C for 24 - 48 hours.

The Wizard DNA cleanup kit (Promega) was used to extract genomic DNA from phage lysates, as previously described (Zorawik et al., 2024) . Genomic DNA libraries were prepared using a NEBNext Ultra II FS kit (New England BioLabs) followed by sequencing using Illumina MiSeq (v3 reagents), yielding ~1.4 and 0.27 million 150-base single-end reads. Raw reads were assembled then checked for completeness using Newbler v2.9 (Miller et al., 2010) and Consed v29 (Gordan and Green, 2013), respectively (Russell, 2018) . Sequencing results and genome characteristics of each bacteriophage are listed in Table 1.

The genomes were auto-annotated using DNA Master v5.23.6 (http://cobamide2.bio.pitt.edu), Glimmer v3.02b (Delcher et al., 2007) , GeneMark v4.28 (Besemer and Borodovsky, 2005) and gene calls manually refined using PECAAN v20221109 (https://pecaan.kbrinsgd.org/index.html), Starterator v462 (https://github.com/SEA-PHAGES/starterator), and Phamerator v539 (Cresawn et al, 2011) . Transmembrane helices were predicted using SOSUI v1.11 (Hirokawa et al, 1998) , TOPCONS v2.0 (Tsirigos et al, 2015) , TMHMM v2.0 (Krogh et al, 2001) , and DeepTMHMM v1.0.24 (Hallgren et al, 2022) . No tRNAs were predicted by ARAGORN v1.2.41 (Laslett and Canback, 2004) or tRNAscanSE v2.0 (Lowe and Chan, 2016) . Putative functions for other predicted genes were made using HHPRED v3.2 (against the PDB_mmCIF70, Pfam- v.36, NCBI Conserved Domain databases) (Altschul et al., 1990) and BlastP v2.10.0 (against the PhagesDB and NCBI nonredundant databases) (Söding et al., 2005). All software were used with default parameters.

BeatusComedenti and Cyan were assigned to cluster AT and subclsuter AZ1, respectively, based on gene content similarity (GCS) of at least 35% to sequenced genomes in the Acinobacteriophage database (https://phagesdb.org/) using the GCS tool at phagesDB (Russel and Hatfull, 2017; Pope et al., 2017) . Like other cluster AT phages, BeatusComedenti encodes a putative tail protein that is longer than its tape measure protein, and an endolysin within its structure and assembly genes, while no immunity repressor or integrase functions could be identified (Klyczek et al., 2017) . Cyan, on the other hand, encodes a serine integrase that is conserved across members of the cluster and is therefore predicted to be a temperate phage. The positioning of the single endolysin encoded by AZ1 phages is not conserved and is found amongst genes encoding DNA metabolism functions in phage Cyan.

Nucleotide sequence accession numbers BeatusComedenti and Cyan, are available at GenBank with Accession No. MN586053 and PQ184793 and the Sequence Read Archive (SRA) No. SRX24892104 and SRX24892113 , respectively.
